# Comparative Neuroanatomy of the Lateral Accessory Lobe in the Insect Brain

**DOI:** 10.3389/fphys.2016.00244

**Published:** 2016-06-23

**Authors:** Shigehiro Namiki, Ryohei Kanzaki

**Affiliations:** Research Center for Advanced Science and Technology, The University of TokyoTokyo, Japan

**Keywords:** command neuron, premotor center, ventral body, lateral lobe, descending neuron

## Abstract

The lateral accessory lobe (LAL) mediates signals from the central complex to the thoracic motor centers. The results obtained from different insects suggest that the LAL is highly relevant to the locomotion. Perhaps due to its deep location and lack of clear anatomical boundaries, few studies have focused on this brain region. Systematic data of LAL interneurons are available in the silkmoth. We here review individual neurons constituting the LAL by comparing the silkmoth and other insects. The survey through the connectivity and intrinsic organization suggests potential homology in the organization of the LAL among insects.

The lateral accessory lobe (LAL) is a neuropil that is highly associated with the central complex (CX). The LAL is thought to facilitate communication between the CX and the motor centers. For example, it is proposed that the LAL receives input from the CX and selects the activity of descending output (Wolff and Strausfeld, 2015). Perhaps due to the deep location and lack of the clear anatomical boundaries, few studies have focused on the LAL. A variety of response properties have been reported in the neurons innervating the LAL in different species: flight-correlated activity in locusts (Homberg, 1994), walking-correlated activity in crickets and moths (Kanzaki et al., 1994; Zorović and Hedwig, 2013), driving backwards walking in flies (Bidaye et al., 2014). The LAL is present in all insect species reported thus far and they seem to have a common set of subdomains, suggesting the ground pattern of the LAL organization. It is not clear whether the neuronal homology is present at individual neuron level.

This paper examines the comparability of individual neurons among different species by summarizing the experimental data available for the LAL. Systematic analysis of neuronal morphology has been performed heretofore only in the silkmoth *Bombyx mori* (Mishima and Kanzaki, 1999; Iwano et al., 2010; Namiki et al., 2014). The large-scale data of neuronal morphology is available in *Drosophila* (Chiang et al., 2011; Jenett et al., 2012; Milyaev et al., 2012; Costa et al., 2013), which cover the entire brain. We compare the organization of the LAL in the silkmoth with *Drosophila* and other insects.

## Anatomy

The CX is defined as a group of four midline neuropils: the protocerebral bridge, the fan-shaped body, the ellipsoid body, and the paired noduli (Ito et al., 2014). We essentially follow the terminology proposed by Insect Brain Nomenclature Working Group, though some other studies describe the LAL also as a part of the CX (Boyan and Reichert, 2011; Shih et al., 2015). To improve comparability among arthropod, another definition is also used: the CX as a group of interconnected neuropils, including the central body, the protocerebral bridge, and the LAL (Richter et al., 2010). Heinze et al. (2013) introduced the term “sun compass neuropils” for the LAL, anterior optic tubercle and the members of the CX because these neuropils are highly interconnected and process compass-related stimuli, such as polarized light (Heinze et al., 2013).

**Figure 1:**
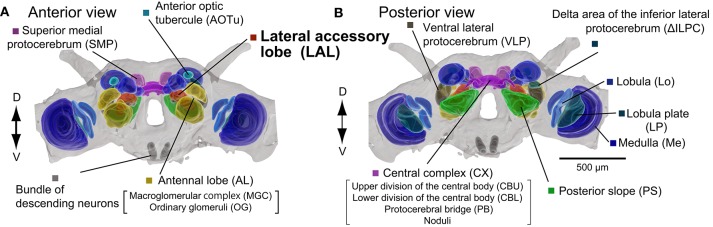
**Organization of the silkmoth brain**. Anterior and posterior views of the silkmoth brain are shown **(A,B)**. Neuropils were segmented based on immunoreactivity of anti-synatotagmin antibody staining in whole-mount sample. Segmentation of unstructured neuropils are not precise because of the lack of clear anatomical boundaries. Images are modified from Namiki et al. (2014). D, dorsal; V, ventral.

**Figure 2:**
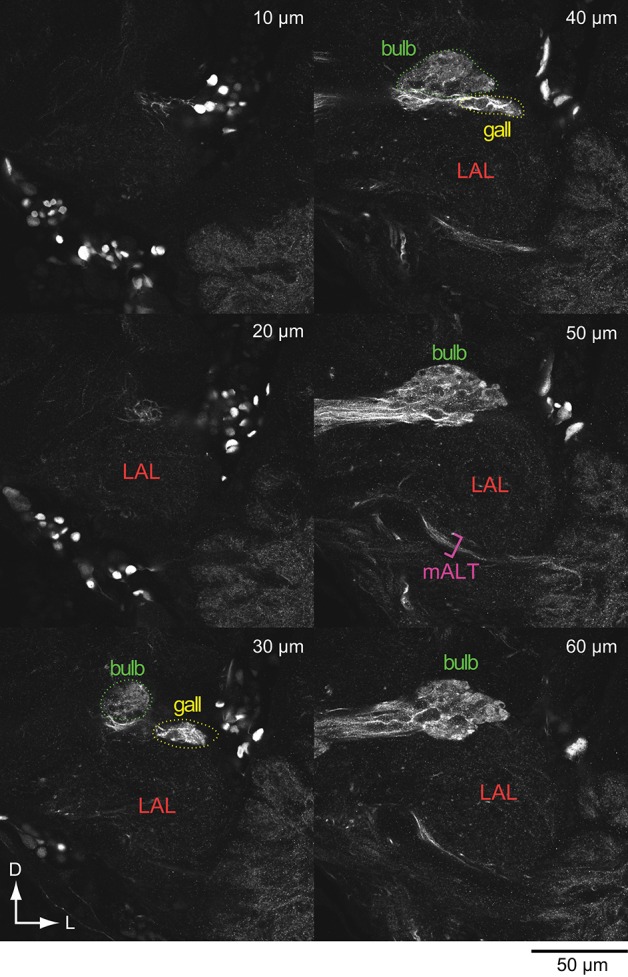
**GABA-like immunoreactivity in the lateral accessory lobe and surrounding area in the silkmoth**. Confocal stacks at the different depth are shown. Anti-GABA antibody was used for the immunoreactivity (wholemount brains; Namiki et al., 2013). D, dorsal; mALT, medial antennal lobe tract; L, lateral.

The LAL is located on the lateral side of the CX in insects (Figure 1; Williams, 1975; Ito et al., 2014). The term “ventral body” is also used in the studies of Diptera. The area surrounding the CX, including the LAL, is called the lateral complex (Ito et al., 2014) or the CX accessory regions (Lin et al., 2013). Comparative neuroanatomy for the organization of the lateral complex is in progress. The LAL and the bulb, a small satellite neuropil, are classified as members of the lateral complex in *Drosophila* (Ito et al., 2014). Figure 2 shows the GABA-like immunoreactivity in the LAL and surrounding region in *Bombyx*. There is a small satellite neuropil located dorsal to the LAL, termed the median olive (Iwano et al., 2010), which shows dense immunoreactivity (Figure 2, termed bulb). This area is rarely connected with the LAL. Based on its position and its immunoreactivity, this region might correspond to the lateral triangle in the monarch butterfly (Heinze and Reppert, 2012), the bulb in *Drosophila* (Ito et al., 2014), the median olive (medial bulb) and the lateral triangle (lateral bulb) in the locust (Heinze and Homberg, 2008; Träger et al., 2008; el Jundi et al., 2014), and the lateral complex in the ant (Schmitt et al., 2015). We denote this neuropil as the bulb, according to the brain nomenclature (Ito et al., 2014; Figure 2). The structure similar to the microglomerular complex (Träger et al., 2008; Seelig and Jayaraman, 2013) is present in the bulb (Figure 2).

A small protruding region in the superior-lateral tip of the LAL is present in the fly, called the gall, and is defined as a part of the LAL (Ito et al., 2014). Similarly, a small sub-region called the anterior loblet, is present in the LAL of the monarch butterfly brain (Heinze and Reppert, 2012). The homologous region exists in *Bombyx* because of the presence of a small region with GABA-like immunoreactivity in the LAL (referred to as gall in Figure 2), and neurons with similar morphology to that of the columnar neurons of the ellipsoid body that project to a similar location as the anterior loblet.

**Figure 3:**
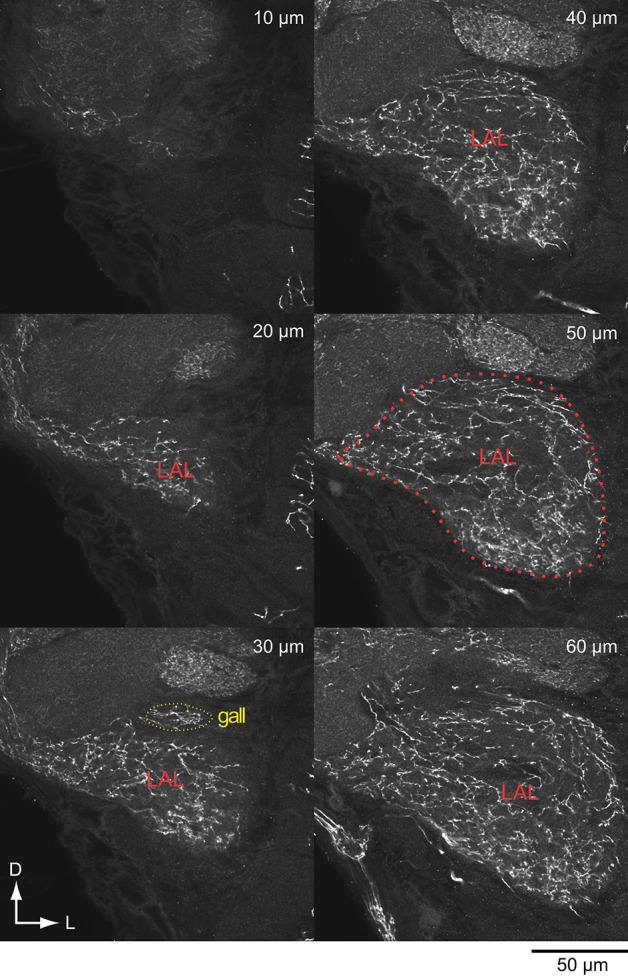
**Serotonin-like immunoreactivity of the lateral accessory lobe and surrounding area in the silkmoth**. Confocal stacks for different depth are shown. Anti-serotonin antibody was used for the immunoreactivity (vibratome sections; Iwano et al., 2010).

The anterior side of the LAL is relatively well-defined, whereas no clear anatomical boundary for the posterior side in most cases (el Jundi et al., 2009; Iwano et al., 2010; Heinze and Reppert, 2012; Ito et al., 2014; Montgomery and Ott, 2015). Practically, the posterior boundary is often defined by the antennal lobe tracts (Iwano et al., 2010; Heinze and Reppert, 2012), which are well-conserved across species (Galizia and Rössler, 2010; Ito et al., 2014). Immunoreactivity often helps to define the anatomical boundary. Figure 3 shows the serotonin-like immunoreactivity of the LAL and surrounding area (Iwano et al., 2010). The antibody-staining covers the entire LAL in *Bombyx* (Figure 3) and might be useful in demarcating the anatomical border of the LAL (Heinze and Reppert, 2012).

Whereas brain regions are usually defined by their anatomy, Chiang et al. (2011) determined brain regions based on the statistical criterion for the clustering of individual neurons, called “local processing units” (Chiang et al., 2011). Using a large-scale data set of single neuron morphology in *Drosophila*, they have identified 41 local processing units. In most cases, the structured neuropils satisfy the criteria for local processing units. The definition is based on individual neuronal morphology, and hence is more functional than the traditional definition based on anatomical landmarks. Through this process, the authors refined the anatomical boundary of the LAL, and defined the inferior dorsofrontal procerebra (IDFP; Chiang et al., 2011), which is composed of four subdomains, including the hammer body, which occupies the largest volume in the LAL, the round body, and the ventral/dorsal spindle bodies.

## Function

The function of the LAL is still incompletely understood. We introduce several examples of the experimental data in different insects, which are helpful to consider the function. Especially we focus on a population of descending neurons (DNs) that originate in the brain and project to the thoracic motor centers.

### Pheromone orientation

Male moths orient to conspecific females by the use of sex pheromones. The circuit within the LAL generates pheromone-evoked persistent firing in the silkmoth (Kanzaki et al., 1994; Namiki et al., 2014). The identical pheromone input can cease persistent activity. The neuronal activity is termed flip-flop, which is a neural signal named after the toggle property. Extracellular recording studies show (1) the correlation between flip-flop neural signals from descending axons and antennal positions and (2) the correlation between antennal positions and turning direction (Olberg, 1983). From these observations, the flip-flop signal is thought to mediate walking commands for pheromone orientation.

**Figure 4:**
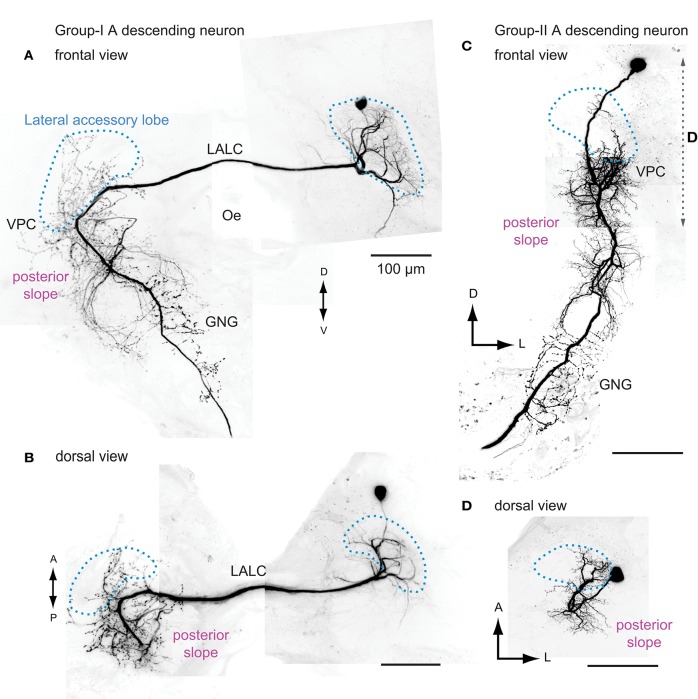
**The morphology of flip-flop descending neurons innervating the lateral accessory lobe in the silkmoth**. Frontal and dorsal views of the group-IA DN **(A,B)** and the group-IIA DN **(C,D)** are shown. Group-IA DN has smooth processes mainly within the ipsilateral LAL and varicose processes in the contralateral LAL, ventral protocerebrum, posterior slope, and the gnathal ganglia **(A,B)**. Group-IIA DN has smooth processes in the ipsilateral LAL, ventral protocerebrum and posterior slope, and varicose processes in the ipsilateral posterior slope, gnathal ganglia **(C,D)**. Outline of the LAL is shown with broken line (*blue*). The range of maximum intensity projection for **(D)** is shown by broken line in **(C)**. GNG, gnathal ganglion; Oe, esophagus; VPC, ventral protocerebram. Images are prepared based on the data used in Mishima and Kanzaki (1999). A, anterior; LALC, lateral accessory lobe commissure; GNG, gnathal ganglia; Oe, esophagus; P, posterior; VPC, ventral lateral protocerebrum.

Three types of DNs that show flip-flop neural signals have been identified so far (Mishima and Kanzaki, 1999; Wada and Kanzaki, 2005; Figure 4). All DNs innervate the LAL. Group-IA DN has smooth processes in the ipsilateral LAL and the varicose processes in the contralateral LAL (Mishima and Kanzaki, 1999), which are the indicators for the postsynaptic and presynaptic terminals (Cardona et al., 2010). Group-IIA and group-IID DNs have smooth processes in the ipsilateral LAL and descend ipsilateral neck connective (Wada and Kanzaki, 2005). The axonal projection in the ventral nervous system is unknown in the silkmoth.

Pheromone information is processed by multiple neural circuits in the silkmoth brain and the pheromone-evoked persistent firing activity is only observed in the neurons innervating the LAL (Namiki et al., 2014), suggesting that the LAL is the site where the flip-flop neural signal is produced.

### Flight-correlated activity

Although the behavioral consequence is not known, the sensory response of LAL DNs are examined in the locust under the tethered flight condition (Homberg, 1994). The flight status is monitored by myographic recordings from the first basaler muscle of the hind wing. The VG3, DN innervating the ipsilateral LAL and descending the contralateral neck connective, shows wind-elicited excitation, which precedes the onset of flight motor activity. The dendritic branch is concentrated in the ventral shell of the LAL. The other ipsilateral LAL DNs also show the flight-preceding activity and innervation mostly to the ventral region of the LAL. Further ascending neurons projecting to the LAL show tonic excitation during flight, and these receive the input from wing proprioreceptors. Based on single cell recording and staining data, the LAL appears to link ascending and descending pathways.

### Phonotactic steering

Female crickets orient toward conspecific males by the use of a calling song. Intracellular recording and staining from brain interneurons reveal the information flow from the ascending neurons via local interneurons toward DNs and indicate the relevance of the LAL for the phonotaxis of the cricket *Gryllus bimaculatus* (Zorović and Hedwig, 2011). Ascending interneurons transmit sound information into the brain. A local interneuron that projects from the axonal area of the ascending interneuron toward the LAL has been reported (Zorović and Hedwig, 2011).

A bilateral LAL DN termed the B-DC1(5) is thought to mediate sensory-motor pathways for phonotaxis (Zorović and Hedwig, 2013). This neuron has smooth, dendrite-like processes in the ipsilateral LAL and the blebby, axon-like processes in the contralateral LAL, and descends on the contralateral side. The morphology is quite similar to the group-IA DN in silkmoths. The depolarization of the B-DC1(5) elicits walking and steering on the contralateral side, and hyperpolarization causes the cessation of walking. The B-DC1(5) response can follow the temporal structure of the male song both in standing and walking conditions, whereas most of the DNs show state-dependent responses, such as gating (Böhm and Schildberger, 1992; Staudacher, 2001). Additionally, unilateral LAL DN termed the B-DI1(1) innervates the ipsilateral LAL and is able to trigger walking, though the effect is less reliable than the B-DC1(5) (Zorović and Hedwig, 2013).

### Obstacle negotiating behavior

Harley and Ritzmann (2010) examined the transition behavior for negotiating obstacles in the cockroach *Blaberus discoidalis*. The authors developed an electrolytic lesioning technique that enabled ablation in a small region, and they examined the effect of the lesion on the behavioral task, including climbing over a block, climbing over/tunneling under a shelf, walking up a wall, and walking in U-shaped track (Harley and Ritzmann, 2010). The authors systematically performed electrolytic lesions within the central complex neuropils and the LAL and evaluated the behavioral abnormality. The lesion within the LAL caused a strong phenotype in most, if not all, obstacle negotiation behaviors, supporting the anatomical observation that the LAL is a major output site of the CX. Additionally, the lesion of the LAL on one side exhibited turning abnormalities in both directions, suggesting the possibility that the turning behavior is not caused by the operation of a single LAL, but rather the coordination of the LAL on both sides is required.

### Backwards walking

Using genetic engineering in *Drosophila melanogaster*, a recent study identified two pairs of neurons for controlling backwards walking, named the moonwalker DN (MDN; Bidaye et al., 2014). When the MDN is activated using thermogenetics, the probability of backwards walking becomes significantly higher, and the silencing of the DNs nearly abolished the movement. These DNs have putative dendritic innervations mainly to the LAL, which is suggested by a synaptic marker. In contrast to the LAL DNs reported in the other insects, MDN innervates the LAL on both sides. The MDN sends projections to leg neuropils in the ventral nervous system on one side. This pattern of innervation is similar to the other ipsilateral LAL DN, aSP3 (Yu et al., 2010), which shows a striking similarity in the morphology in the brain to the group-II DNs of the silkmoth.

### Polarized light processing

Locusts are known for its long-distance migration. They use the polarized light as a sky-compass information for navigation. The neuronal pathway of the polarized light processing has been investigated in detail (Heinze, 2014). Columnar neurons of the CX respond to sky compass signal in locusts (Vitzthum et al., 2002; Heinze and Homberg, 2007), butterflies (Heinze and Reppert, 2011), and beetles (el Jundi et al., 2015). Because the columnar neurons project to the LAL, the area might be relevant for polarized light processing. Polarized sensitive neurons that can be postsynaptic to the CX neurons have been described (Heinze and Homberg, 2009). One of these neurons connects the ipsilateral LAL to the contralateral triangle and show the polarization opponency, suggesting the polarized light processing in the LAL. The LAL-pPC neuron, projects to the posterior protocerebrum, which may correspond to the posterior slope in the silkmoth and the posterior slope/inferior bridge in *Drosophila*. This neuron might supply a polarization-sensitive descending neurons in the locust (Träger and Homberg, 2011).

Overall, these examples suggest the LAL function on locomotion, such as steering toward left or right, and moving forward or backward. Steering-related functions appear to be rare in the other types of DNs, which do not innervate to the LAL, including middle leg contractions for fast escape in the giant fiber (von Reyn et al., 2014), triggering courtship behavior in the pIP10 (von Philipsborn et al., 2011) and the P2b (Kohatsu et al., 2011), grooming in antennal DNs (Hampel et al., 2015), leg motion in a dopaminergic DN in *Drosophila* (Tschida and Bhandawat, 2015), flight initiation in TCG in locusts (Bicker and Pearson, 1983), and song generation in the B-DC-3 of crickets (Hedwig and Heinrich, 1997). In this respect, it would be interesting to identify whether the deviation sensitive DNs, such as the DCI and the PI(2)5 in locusts (Hensler, 1988; Hensler and Rowell, 1990) that are assumed to generate steering responses, have dendritic innervation into the LAL.

## Neuronal morphology

The LAL is connected to various regions of the protocerebrum (Strausfeld et al., 1998; Namiki et al., 2014). In this section we introduce the morphology of LAL interneurons according to the connectivity. We also examine the comparability in their morphology, which suggests homology of the LAL among insects.

### Central complex

There are dense connections between the fan-shaped body/protocerebral bridge and the LAL (Shih et al., 2015). Detailed morphology of individual neurons has been reported in *Drosophila*, the desert locust, and the monarch butterfly (Heinze and Homberg, 2008; Heinze et al., 2013; Lin et al., 2013; Wolff et al., 2015). The cytoarchitecture and the morphology of individual neurons seem to be conserved. The LAL receives input from populations of columnar neurons connected to the ellipsoid body via eb-pb-vbo (EIP)/CL1a neurons, protocerebral bridge via the pb-eb-idfp (PEI)/CL1b,d neurons, and the fan-shaped body and protocerebral bridge via the pb-fb-idfp (PFI)/CPU1,2 neurons (Heinze and Homberg, 2008; Heinze et al., 2013; Lin et al., 2013). The basic neuronal components are similar in other insects, including the cricket (Schildberger, 1983), honeybee (Homberg, 1985), beetle (Wegerhoff et al., 1996), and silkmoth (Namiki et al., 2014) (Supplementary Figure 1). There are connections between the LAL and circuit components of the CX that travel in opposite directions and form functional loops (Lin et al., 2013; Shih et al., 2015).

### Anterior optic tubercle

The anterior optic tubercle is the most prominent anterior optic focus in the protocerebrum. There are parallel processing pathways for processing polarized light in the locust. The upper unit of the anterior optic tubercle supplies the LAL, whereas the lower unit supplies the bulb (median olive and lateral triangle; Homberg et al., 2003). Connections between the anterior optic tubercle and the LAL have been reported in *Drosophila* (Yang et al., 2013; e.g., Cha-F-600143, Cha-F-000252, fru-M-800104, Gad1-F-300056), honeybee (Mota et al., 2011), bumblebee (Pfeiffer and Kinoshita, 2012), and silkmoth (Namiki et al., 2014). This pathway underlies sky-compass navigation that uses polarized light in insects (Homberg et al., 2011).

### Superior medial protocerebrum

The superior medial protocerebrum is an unstructured neuropil located in the dorsal medial part of the protocerebrum. The brain region is identified as the relay station between the lateral protocereberum and the LAL through a pheromone processing pathway in the silkmoth (Namiki et al., 2014). Dye injection into the LAL labels this region. The neurons projecting from superior medial protocerebrum to the contralateral LAL have been identified (Supplementary Figure 2) (Namiki et al., 2014). A connectome study suggested the presence of this connectivity in *Drosophila*. The superior dorsofrontal protocerebrum, which roughly corresponds to the superior medial protocerebrum, is highly connected with the IDFP (mostly overlap to the LAL; Shih et al., 2015), and interneurons that connect these regions are present (e.g., Cha-F-000105, TH-F-200089, TH-F-200092, VGlut-F-000104; Chiang et al., 2011). The connection between the superior medial protocerebrum and the LAL are also indicated by studies based on clonal units (Ito et al., 2013; Yang et al., 2013). The dorsal-anterior-lateral neurons, which are relevant to memory retention, connect the superior dorsofrontal protocerebrum and IDFP (Chen et al., 2012). The F1 neurons, which are a neuronal population involved in visual pattern memory for contour orientation (Liu et al., 2006; Li et al., 2009), innervate LAL, fan-shaped body and the superior medial protocerebrum. The neurons connecting the LAL and the superior medial protocerebrum have also been reported in the flesh fly (Phillips-Portillo and Strausfeld, 2012) the desert locust (Homberg et al., 2003) and the monarch butterfly (Heinze et al., 2013).

The brain region is characterized by several unique features. The interneurons innervating this region respond to multimodal sensory stimulation and often show spontaneous burst activity in the silkmoth (Supplementary Figure 3). Connectomics studies identify the superior medial protocerebrum as a hub in the whole brain network in *Drosophila* (Ito et al., 2013; Shih et al., 2015). The entire population of output neurons from the mushroom body lobes has been described in *Drosophila*, and the majority of them supply the superior medial protocerebrum (Aso et al., 2014). These anatomical data suggest the importance of this brain region. Neuronal activity preceding walking is reported in the crayfish (Kagaya and Takahata, 2011). The neuron has the dendritic branch in the area close to the central body, which locates potentially similar to the superior medial protocerebrum in insects.

### Lobula complex

**Figure 5:**
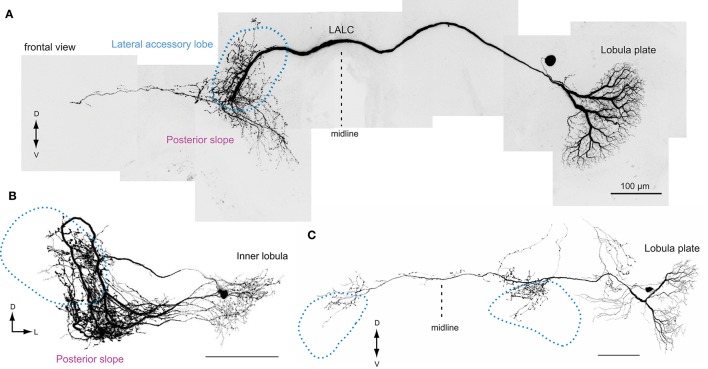
**The morphology of lobula complex widefield cells supplying the lateral accessory lobe in the silkmoth**. **(A)** This neuron has smooth process in the ipsilateral lobula plate and varicose processes onto the contralateral LAL and posterior slope. The varicose processes are mainly concentrated onto the upper division of the LAL. **(B)** A neuron with smooth branches in the inner lobula and has varicose processes in the ipsilateral LAL and the posterior slope. **(C)** A neuron with smooth branches in the lobula plate and varicose processes to the bilateral LALs. In each case, the projection within the LAL are biased to the upper division. Images are modified from Namiki et al. (2014). LALC, lateral accessory lobe commissure.

Namiki et al. (2014) identified a pathway from the lobula complex (Figure 5) directly innervating the LAL. The wide-filed dendritic branches in the lobula plate is reminiscent of the lobula-plate tangential cells in Diptera (Hengstenberg et al., 1982). Optic lobe projection neurons from both the medulla and the lobula complex project to the posterior slope in *Bombyx*. However, only the neurons from the lobula complex have additional processes onto the LAL. The direct inputs from the lobula complex to the LAL are also present in *Drosophila* (e.g., VGlut-F-800153 for ipsilateral, VGlut-F-300218 for contralateral LAL; data available from FlyCircuit Database; Chiang et al., 2011). The leukokinin-immunoreactive neurons connecting the anterior lobe of the lobula and the LAL have been reported in locusts (Homberg et al., 2003).

The direct input from the lobula plate to the LAL might enable integration of olfactory and visual information. Walking activity evoked by sex pheromones is modulated by the presence of the optic flow, especially in the surge phase, in the silkmoth (Pansopha et al., 2014). Silkmoths utilize visual information to modify locomotor commands to adapt to perturbations in the sensory-motor feedback gain (Ando et al., 2013). Although there is no experimental evidence, the identified direct pathway to the LAL might underlie this behavior.

### Thoracic motor centers

**Figure 6:**
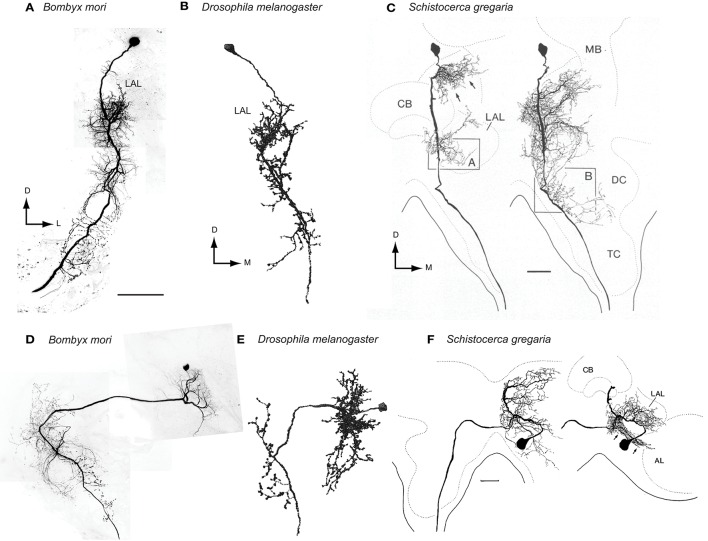
**Comparison of neuronal morphology of the LAL bilateral neurons across species. (A)** Ipsilateral LAL DN of the silkmoth, *Bombyx mori* (Mishima and Kanzaki, 1999). **(B)** Ipsilateral LAL DN of the fruit fly *Drosophila melanogaster* (VGlut-F-200356, FlyCircuit Database; Chiang et al., 2011). The neuron has smooth processes in the ipsilateral LAL, vest, wedge and superior posterior slope and descend the ipsilateral neck connective. **(C)** Ipsilateral LAL DN of the desert locust, *Schistocerca gregaria* (Homberg, 1994). **(D)** Bilateral LAL DN of the silkmoth, *Bombyx mori* (Mishima and Kanzaki, 1999). **(E)** Bilateral LAL DN of the fruit fly *Drosophila melanogaster* (VGlut-F-500726, FlyCircuit Database; Chiang et al., 2011). This neuron has smooth processes in the ipsilateral LAL, epaulete, vest, inferior posterior slope and superior posterior slope and varicose processes in the contral lateral vest, inferior posterior slope and superior posterior slope. **(F)** Bilateral LAL DN of the desert locust, *Schistocerca gregaria* (Homberg, 1994). Scale bars = 100 μm. AL, antennal lobe; CB, central body; DC, deep deutocerebrum; LAL, lateral accessory lobe; M, medial; MB, mushroom body; TC, tritocerebrum.

There is similarity in the morphology of DNs innervating LALs among insect species (Figure 6). For example, the characteristic morphological features of group-II DNs in *Bombyx* are: (1) cell bodies belong to the cluster located on the anterior surface beside the anterior optic tubercle, (2) they descend the ipsilateral side of the neck connective, and (3) they have smooth processes in the LAL. The neurons that meet these morphological features have been reported in other species, including the sphinx moth *Manduca sexta* (Kanzaki et al., 1991a), the cricket *Gryllus bimaculatus* (Zorović and Hedwig, 2013), the locust *Schistocerca gregaria* (Homberg, 1994), and the fruit fly *Drosophila melanogaster* (Yu et al., 2010). This observation suggests the homology of neuronal morphology for at least some types of DNs. In this respect, testing the axonal projections into the ventral nervous system would be interesting, but has not been studied thus far. Similarly, some bilateral LAL DNs also share their morphological features: group-IA DNs in moths (Figure 6D), VGlut-F-500726 in *Drosophila* (Figure 6E), VG3 in locusts (Figure 6F), and B-DC1(5) in crickets (Homberg, 1994; Mishima and Kanzaki, 1999; Zorović and Hedwig, 2011). Potentially similar neurons are present in ants (Yamagata et al., 2007) and dragonflies (Olberg, 1986).

The bilateral LAL DNs have smooth processes and putative dendritic regions in one side of the LAL in most cases. An exception is the MDN that has dendritic innervations in bilateral LALs (Bidaye et al., 2014). MDN-like cells might be implemented in other insect species that show backwards walking, such as the stick insect (Graham and Epstein, 1985). The silkmoth does not show backwards walking at least in normal condition. Even though intracellular recording on a continuous basis has been performed, which targets the LAL over the past two decades, we have never observed this neuron type in the silkmoth.

### Posterior slope

Although several lines of evidence indicate function of the CX on behavioral control such as place learning (Ofstad et al., 2011), spatial navigation (Neuser et al., 2008; Seelig and Jayaraman, 2015; el Jundi et al., 2015), locomotor control (Martin et al., 2015), the information flow from the CX and thoracic motor centers is still unclear. Because the CX might have very few or no direct descending outputs (Staudacher, 1998; Okada et al., 2003; Cardona et al., 2009; Hsu and Bhandawat, 2016), some other parts of the brain must be involved in relaying the command information. The LAL is the primary candidate because of its dense connections with the CX and the several examples of DNs that control locomotion (Zorović and Hedwig, 2013; Bidaye et al., 2014). The number of DNs innervating the LAL, however, is much smaller than that in other parts of the brain, such as the posterior slope, lateral protocerebrum, and gnathal ganglion (Strausfeld et al., 1984; Ito et al., 1998; Okada et al., 2003). In the silkmoth, we estimate that approximately 10 DNs innervate the LAL on each side. One possibility is that such a small number of neurons enable a versatile behavioral repertoire. Another possibility is that another brain region, that is downstream to either the CX and/or the LAL, might relay information to the thoracic motor centers.

**Figure 7:**
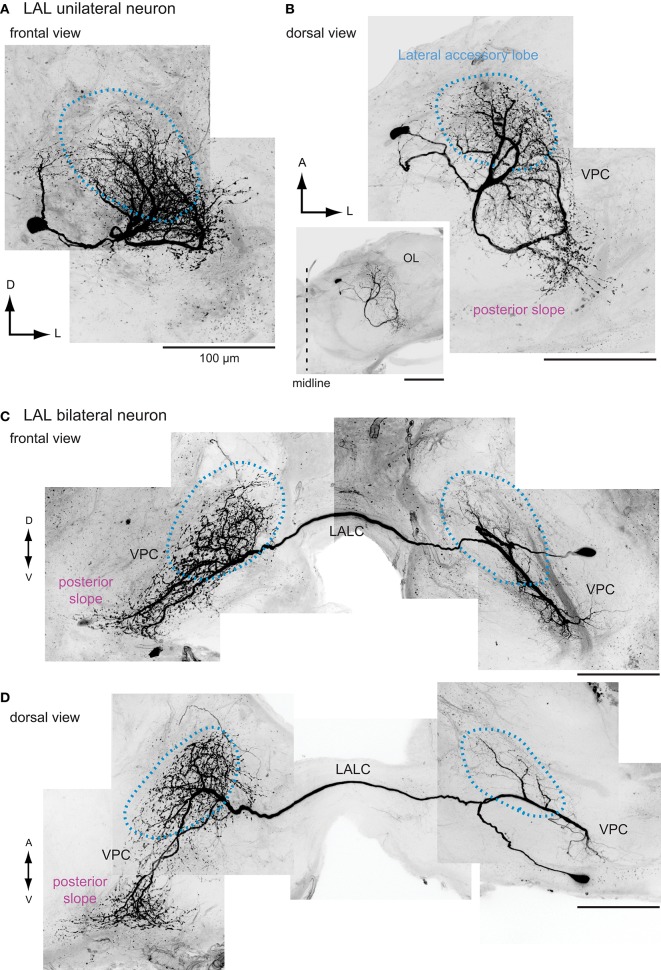
**The morphology of interneurons innervating the lateral accessory lobe in the silkmoth**. Frontal and dorsal views of unilateral LAL interneurons **(A,B)** and bilateral LAL interneurons **(C,D)** are shown. Images are prepared based on the data used in Iwano et al. (2010). LALC, lateral accessory lobe commissure; VPC, ventral protocerebrum.

We postulate that the posterior slope connects the LAL with the major population of DNs and then transmits the information to the thoracic motor centers. The posterior slope is the inferior part of the posterior brain, where extensive arborizations of descending and ascending neurons are observed (Strausfeld, 1976; Ito et al., 2014). Lobula plate neurons supplies this region, and hence is thought to be involved in the processing of motion cues (Strausfeld and Bassemir, 1985; Paulk et al., 2009; Borst, 2014). The posterior slope contains the largest number of DNs in the cerebral ganglia. In all of the species studied thus far, the posterior-ventral part of the brain is densely labeled by backfilling from the neck connective (Kanzaki et al., 1994; Staudacher, 1998; Okada et al., 2003; Cardona et al., 2009; Hsu and Bhandawat, 2016). Additionally, the posterior slope has connections with the LAL, which might be bidirectional. Namiki et al. (2014) reported the connection with the LAL by injecting the fluorescent dye into the posterior slope. From the neuronal morphology obtained by intracellular staining, there are candidate neurons for these connections. For example, a subpopulation of the LAL interneurons identified so far in *Bombyx* actually have innervations to the posterior slope (Figure 7). About half of the LAL interneurons have varicose processes in the posterior slope (40%, *n* = 20 for bilateral neurons; 55%, *n* = 9 for unilateral neurons; Namiki et al., 2014). A group of unilateral interneurons connects the LAL and the posterior slope (Iwano et al., 2010). Neurons connecting the LAL and the posterior protocerebrum have been reported in the locust (Heinze and Homberg, 2009) and butterfly (Heinze and Reppert, 2012).

Next, we considered the possibility that the DNs themselves transmit information from the LAL to the posterior slope. A subpopulation of bilateral DNs have smooth processes in the LAL in the ipsilateral side and varicose processes in the contralateral side that might mediate the information flow from the posterior slope to the LAL (Figure 4). Group-I DNs, all of which show bilateral innervations, have varicose terminal processes in the contralateral posterior slope, and all of the ipsilateral LAL DNs studied so far have varicose terminals in the ipsilateral posterior slope (Mishima and Kanzaki, 1999; Namiki et al., 2014). The putative homologous neurons of the *Bombyx* group-I DNs in *Drosophila* show similar morphological feature. They also have additional innervations in the posterior slope (e.g., VGlut-F-500726; Figure 6E, VGlut-F-000150, and fru-F-100073; FlyCircuit Database; Chiang et al., 2011). These anatomical connections suggest a large degree of interplay between these two circuits.

### Other regions

The LAL is also connected with other neuropils in the protocerebrum, such as the ventrolateral protocerebrum in the moth (Pfuhl et al., 2013), locust (Homberg, 1994), and *Drosophila* (e.g., Gad1-F-000101, fru-M-300049; Chiang et al., 2011). This region contains descending output (Milde and Strausfeld, 1990; Okada et al., 2003). Although the number of neurons involved in this connection might be small, this pathway might also underlie the transmission of command from the CX (Strausfeld and Hirth, 2013).

Although the number of neurons is small, connections with the mushroom body are present in *Drosophila* (Ito et al., 1998). The connection between the LAL and the medial lobe of the mushroom body has been described in the blowfly *Calliphora erythrocephala* (Strausfeld, 1976). A neuron connecting the LAL and the mushroom body pedunculus has been reported in the cockroach, which are sensitive to mechanosensory stimuli (Strausfeld and Li, 1999).

## Intrinsic organization

The LAL appears to have modular organization in the silkmoth (Namiki et al., 2014). We here review the intrinsic organization in the silkmoth by comparing the neuronal morphology with other insects.

**Figure 8:**
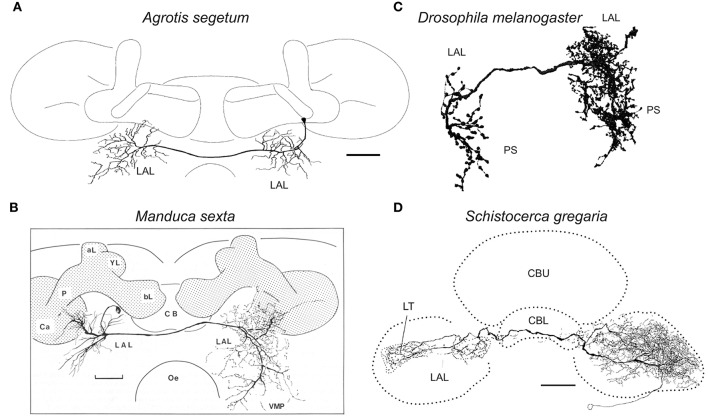
**Comparison of neuronal morphology of the LAL bilateral neurons across species. (A)** LAL bilateral neuron of the turnip moth *Agrotis segetum* (Lei et al., 2001). **(B)** LAL bilateral neuron of the sphinx moth *Manduca sexta* (Kanzaki et al., 1991b). **(C)** LAL bilateral neuron of the fruit fly *Drosophila melanogaster* (Fru-M-200330, FlyCircuit Database; Chiang et al., 2011). The neuron has smooth processes in the ipsilateral LAL, epaulete, wedge, vest and superior posterior slope and varicose processes in the contralateral LAL, inferior posterior slope and superior posterior slope. **(D)** LAL bilateral neuron of the desert locust, *Schistocerca gregaria* (Heinze and Homberg, 2009). Scale bars, 50 μm for **(A)**, 100 μm for **(B,D)**. aL, alpha-lobe of the mushroom body; bL, beta-lobe of the mushroom body; Ca, calyx of the mushroom body; CB, central body; CBL, lower division of the central body; CBU, upper division of the central body; LAL, lateral accessory lobe; LT, lateral triangle; Oe, esophagus; P, pedunculus of the mushroom body; PS, posterior slope; VMP, ventral-medial protocerebrum.

The interneurons of the LAL are classified into two groups: unilateral neurons innervating one side of the LAL and bilateral neurons innervating both sides of the LAL (Figure 7). One prominent feature in circuit organization is the dense connection between both hemispheres, which contains bilateral neurons (Homberg et al., 1987; Homberg and Hildebrand, 1989, 1991; Breidbach, 1990; Müller et al., 1997; Dacks et al., 2006). In the silkmoth, about 60 fibers run through the LAL commissure, a bundle of bilateral neurons connecting the LALs on both sides. This neuronal population is thought to have a crucial role on generating walking command (Kanzaki, 1997). Among these, many neurons show GABA-like immunoreactivity (Iwano et al., 2010). Additionally, there are two pairs of bilateral neurons in the LAL with serotonin-like immunoreactivity, which are present also in other species including Lepidoptera, Coleoptera, and Diptera (Dacks et al., 2006). A population of LAL bilateral neurons has been identified by clustering analysis of FlyCircuit Database (Cluster 31 in supercluster XII; Chiang et al., 2011; Costa et al., 2014). The anatomy of single neuron morphology of the LAL bilateral interneurons has been reported in moths including *Heliothis virescens* (Pfuhl and Berg, 2007), *Agrotis segetum* (Figure 8A; Lei et al., 2001), and *Manduca sexta* (Figure 8B; Kanzaki et al., 1991b), fruit flies (Figure 8C; Hanesch et al., 1989; Chiang et al., 2011), crickets (Zorović and Hedwig, 2011), and locusts (Figure 8D; Müller et al., 1997; Heinze and Homberg, 2009). A similar neuron is present in the malaria mosquito *Anopheles gambiae* (Ignell et al., 2005).

The LAL is classified into two subdivisions that are delineated by the LAL commissure that is the prominent bundle connecting the bilateral LALs: upper division and lower division (Iwano et al., 2010). The LAL bilateral neurons can be classified into two morphological classes based on the degree of neurite innervation into the upper and lower divisions (Supplementary Figure 4). The same morphological feature is observed in *Drosophila* (VGlut-F-800201 for the lateral side, VGlut-F-700549 and VGlut-F-800001 for the medial side of the IDFP) and locust (Homberg, 1994). The interneurons with biased innervations to the lower division of the LAL exhibit activity with a longer duration in response to sex pheromones (Supplementary Figure 5) (Namiki et al., 2014). This suggests the functional difference between the upper and lower divisions.

The inputs from the CX terminate in specific sub-regions within the LAL (Heinze et al., 2013; Lin et al., 2013; Namiki et al., 2014), suggesting the presence of a functional module within the LAL. Most of the input from the CX converges onto the upper division of the LAL in *Bombyx* (Supplementary Figure 6) (Namiki et al., 2014), the ventral LAL in monarch butterfly (Heinze et al., 2013), and the dorsal shell of the LAL in locust (Heinze and Homberg, 2008). The columnar neurons project to the lateral side of the IDFP or LAL in the *Drosophila* (Lin et al., 2013; Wolff et al., 2015). In terms of connectivity, the ventral shell might be homologous to the monarch butterfly's dorsal LAL and the upper division of the LAL in *Bombyx* and *Drosophila*. The lobula complex also supplies biased inputs to the LAL (Supplementary Figure 6) and the same morphological feature is observed in *Drosophila* (Namiki et al., 2014). The projection of a population of dopaminergic PPM3 neurons in *Drosophila* seems to be biased toward the lateral side of the LAL (Nässel and Elekes, 1992; Ueno et al., 2012; Alekseyenko et al., 2013).

The dendritic innervation of LAL DNs is biased to the lower division (Supplementary Figure 7, right). For example, group-IA DN has small dendritic field in the upper division and much more innervation in the lower division, and Group-IID DN shows almost no innervation to the upper division (Supplementary Figure 7, left). Overall, the relative volume of innervations in the lower division is significantly more than those in the upper division (Namiki et al., 2014).

Putative homologous neurons of *Bombyx* group-I DNs in *Drosophila* show similar features (VGlut-F-500726, VGlut-F-000150; FlyCircuit Database; Chiang et al., 2011). Their neurite innervations within the LAL are more toward the medial side of the IDFP. This morphological feature seems to be obvious in other LAL DNs such as the MDN, which controls walking direction (Bidaye et al., 2014).

**Figure 9:**
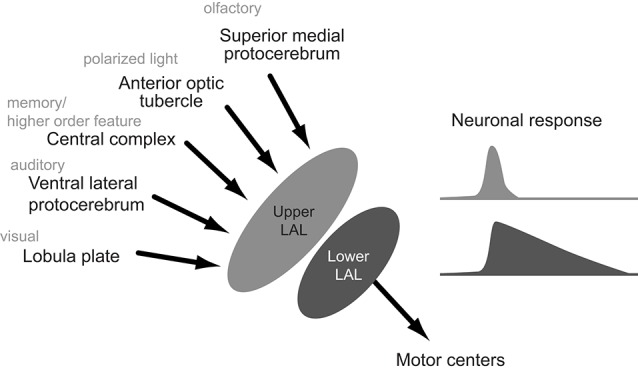
**Schematics of the hypothesized organization of the lateral accessory lobe**. Inputs from the protocerebram converge onto the upper division and the descending output originates the lower division. Neuronal response to the sex pheromone, which trigger the locomotion, is longer for neurons innervating the lower division.

From these anatomical observations, we propose the modular organization of the LAL is common across insects. The upper division integrates the information from multiple protocerebral regions in addition to the CX, while the lower division produces the premotor signal output via DNs (Figure 9).

## Conclusion

We reviewed the neuronal components of the LAL in the silkmoth and described the neurons with similar morphology in *Drosophila* and other insects. There are plentiful examples for their potential homology at the level of individual neurons, suggesting the presence of a ground pattern organization. Insects adapt to various environments in different ways, but the same basic design of the nervous system may underlie diverse behavioral repertoire. Expanding the application of a comparative neurobiological approach provides a powerful clue to explore these mechanisms.

## Author contributions

All authors listed, have made substantial, direct, and intellectual contribution to the work, and approved it for publication.

### Conflict of interest statement

The authors declare that the research was conducted in the absence of any commercial or financial relationships that could be construed as a potential conflict of interest.
