# Supplementary material for: Comparative Neuroanatomy of the Lateral Accessory Lobe in the Insect Brain
Source: Front Physiol. 2016 Jun 23;7:244. doi: 10.3389/fphys.2016.00244 (PMC4917559; doi:10.3389/fphys.2016.00244)
Supplement: Supplementary file 1 [file DataSheet1.docx]

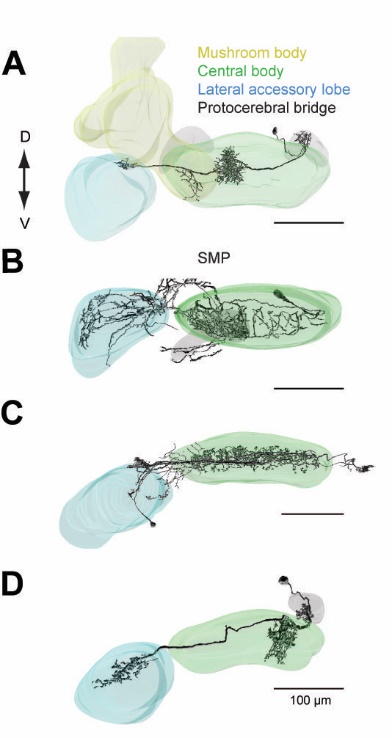


**Supplementary Figure 1| Morphology of neurons connecting the central complex and the lateral accessory lobe in the silkmoth. (A)** Columnar neuron connecting protocerebral bridge, central body lower division, mushroom body medial lobe and the LAL. **(B)** Tangential neuron connecting the central body upper division, protocerebral bridge, and the LAL. **(C)** Tangential neurons connecting the central body lower division and the LAL. **(D)** Columnar neuron connecting the protocerebral bridge, central body upper division, and the LAL. Images are modified from Namiki et al. 2014.


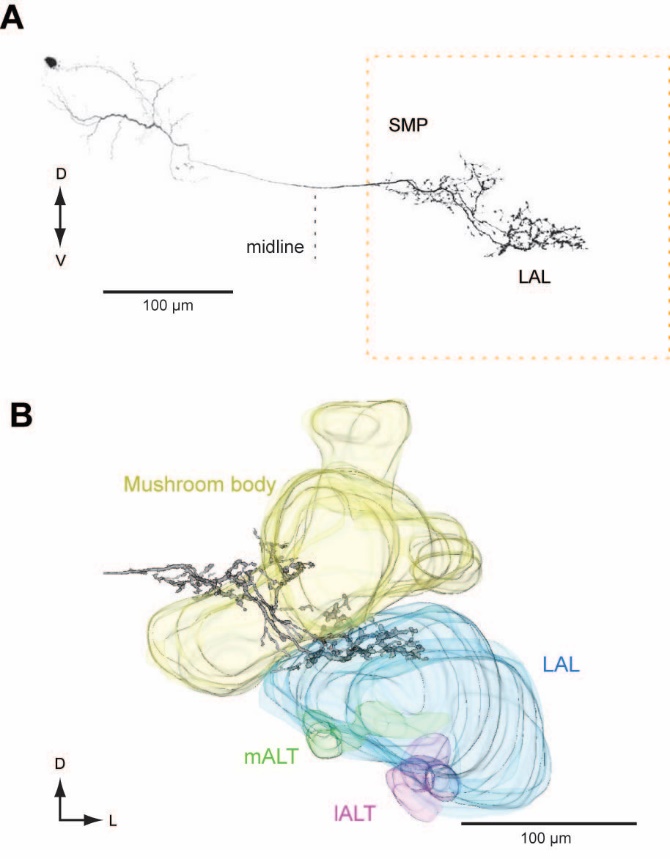


**Supplementary Figure 2 | Morphology of a neuron connecting the superior medial protocerebrum and the LAL in the silkmoth. (A)** Confocal image of the projection neuron from the superior medial protocerbrum. The neuron has smooth processes in the ipsilateral superior medial protocerebrum and varicose processes in the contralateral superior medial protocerebrum and the LAL. **(B)** Reconstructed neurite innervation of the neuron shown in **A**. Images are prepared based on the data used in Namiki et al. 2014. mALT, medial antennal lobe tract; lALT, lateral antennal lobe tract.


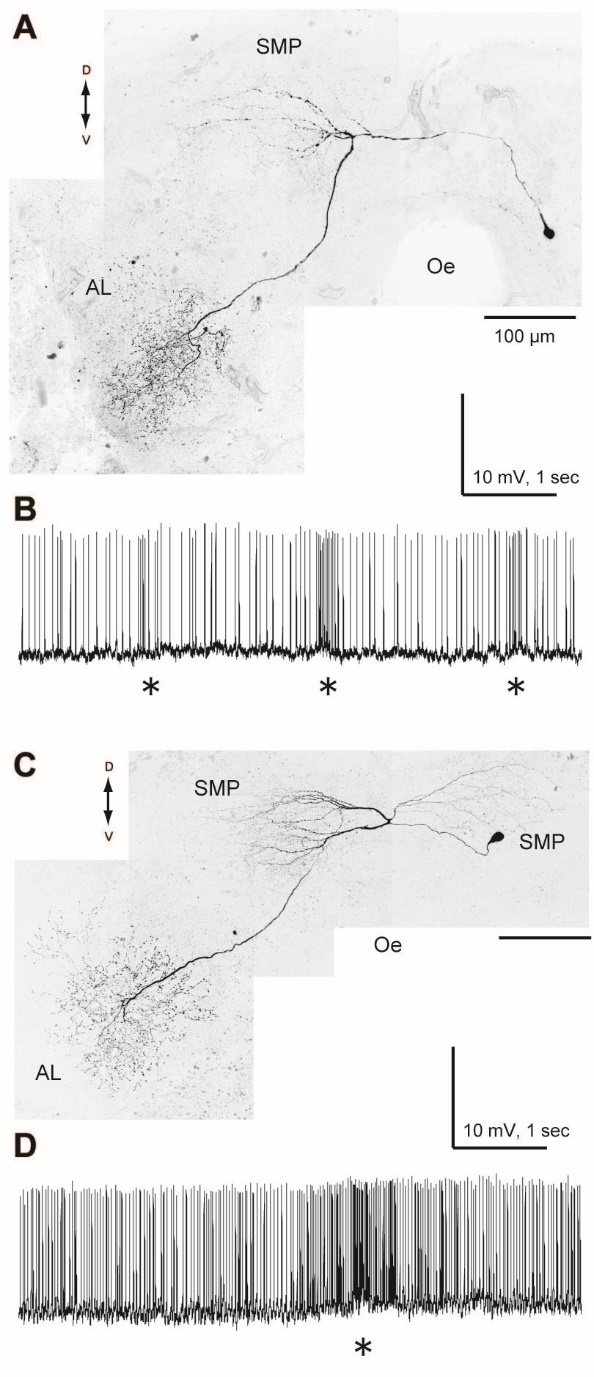


**Supplementary Figure 3 | Physiology and morphology of the neuron innervating the superior medial protocerebrum in the silkmoth.** Two examples of morphology **(A,C)** and physiology **(B,D)** are shown. Spontaneous activity are recorded by intracellular recording technique with glass microelectrode. These neurons show spontaneous burst-like activity marked by asterisks. These neurons have smooth processes in the superior medial protocerebrum and the varicose processes in the contralateral antennal lobe. Images are prepared based on the data used in Namiki et al. 2014. AL, antennal lobe; Oe, oesophagus; SMP, superior medial protocerebrum.


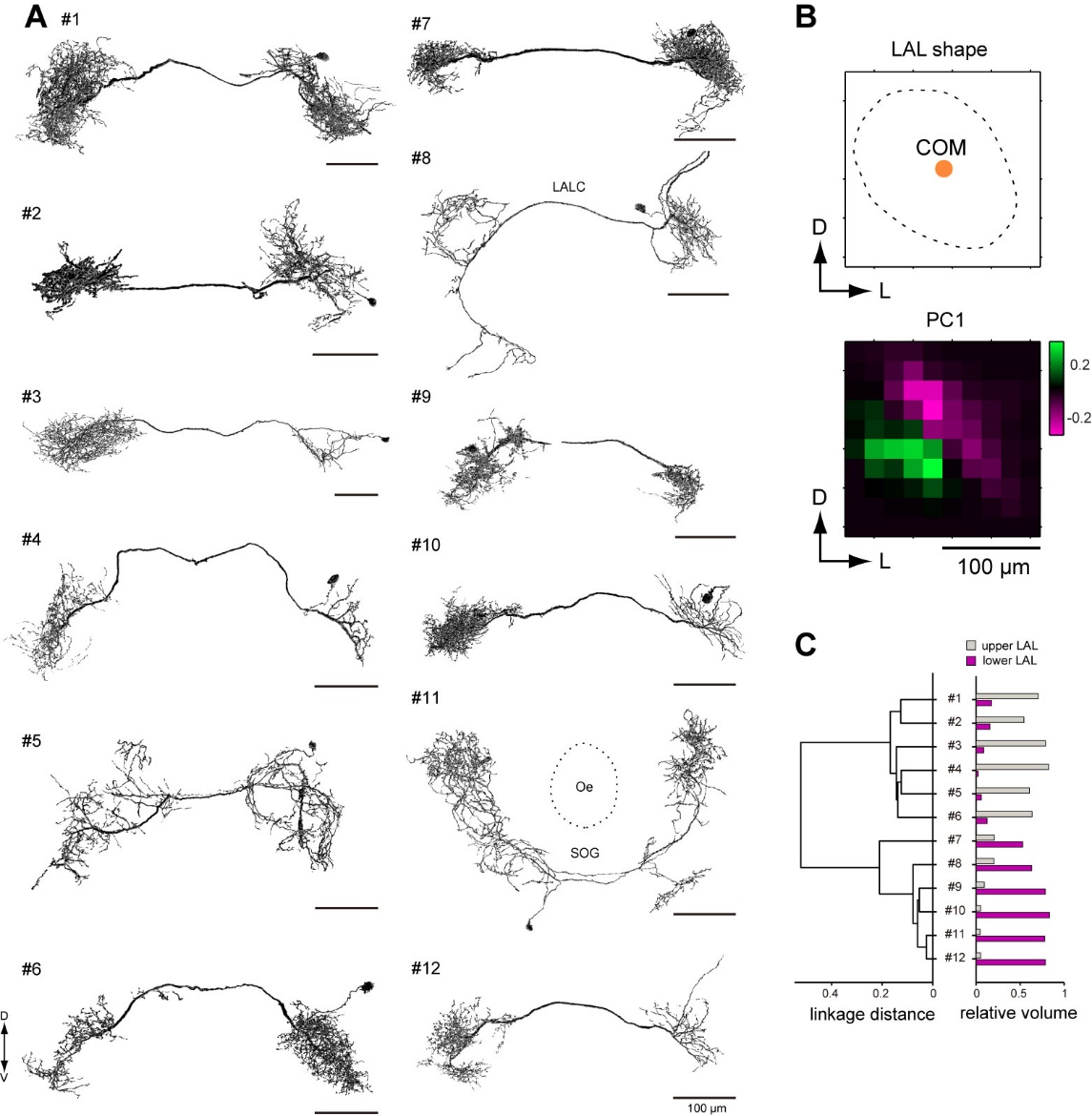


**Supplementary Figure 4 | Classification of lateral accessory lobe bilateral neurons based on dendritic innervaton in the silkmoth.** **(A)** Morphology of the LAL bilateral neurons. The neuron types were noted based on the cell body position and neurite innervation. The areas in which the majority of terminals showed smooth processes and varicose processes are shown with green and magenta, respectively. Cell body location of #12 is shown by an asterisk. **(B)** Classification of the LAL volume based on the innervation by LAL bilateral neurons shown in **A**. Individual data are compared in the coordinate adjusted based on the center of mass of the LAL (*top*). The neurite density is analyzed by principal component analysis for neurite volume of individual neurons (500 voxels×12 neurons). The value of the first principal component is shown (*bottom*). The voxels with positive and negative value mostly correspond to the lower and upper division, respectively. **(C)** Clustering of LAL bilateral neurons based on the density of smooth processes (*left*). Relative volumes of the neurites of LAL bilateral neurons in the upper (grey) and lower divisions of the LAL (magenta) are shown (*right*). The number in the vertical axis corresponds to the numbers in column a. Images are prepared from the data used in Namiki et al. 2014.


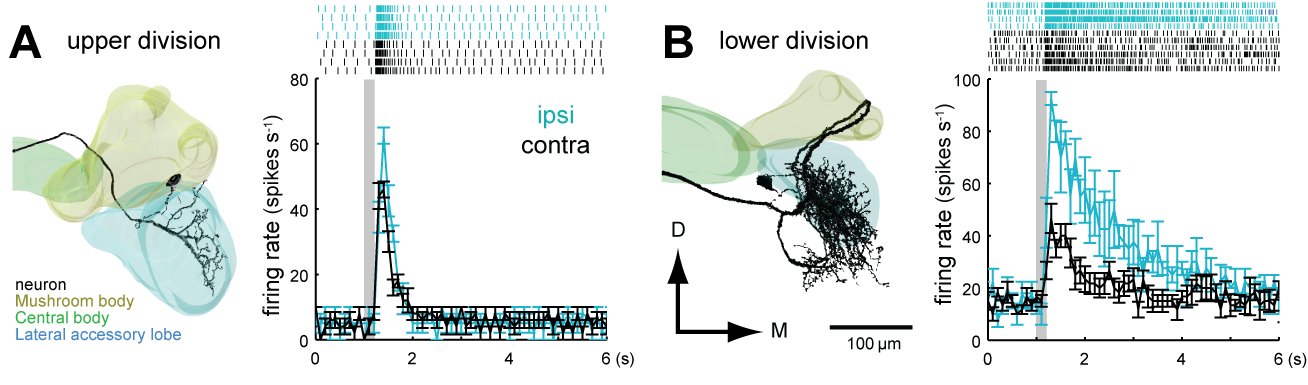


**Supplementary Figure 5 | Neurons innervating the lower LAL exhibited longer activity duration in the silkmoth.** The data of neurons innervating the upper division (**A**) and lower division of the LAL are shown (**B**). The morphology of putative dendritic location is shown in left panel. The shape of neuropils are shown with color. The pheromone-triggered response are shown (Spike timing in *upper right*, mean firing rate in *bottom right*). The gray box represent the time period of pheromone presentation. The activity in response to the ipsilateral and contralateral antenna are shown with blue and black, respectively. Images are modified from Namiki et al. 2014.


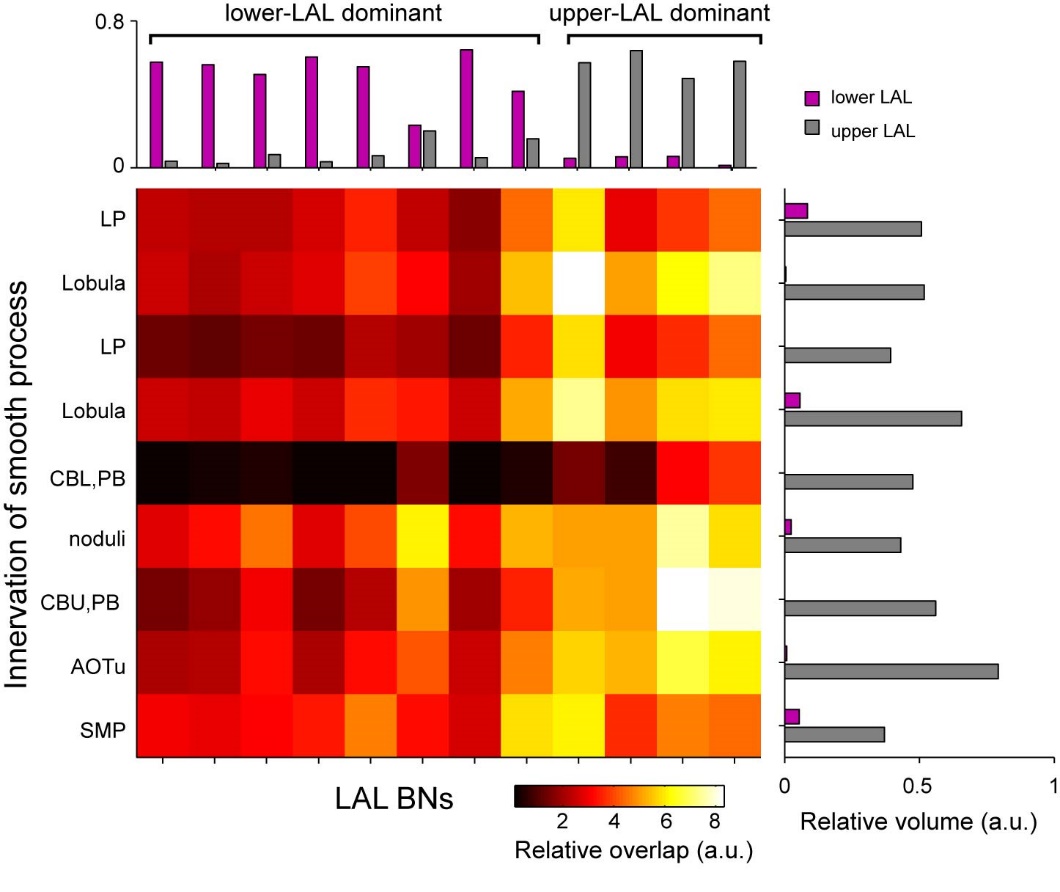


**Supplementary Figure 6| Overlap of the innervation between the LAL input neurons and the LAL bilateral neurons in the silkmoth.** The color matrix shows the overlap of the neurite density for each neuron pair shown by row (input neurons to the LAL) and column (LAL bilateral neurons). The relative volume of the innervation to the lower (*magenta*) and upper divisions of the LAL (*gray*) is shown by bar graph. The LAL input neurons prefer to innervate the upper division (*gray*) and the upper LAL dominant type of bilateral neurons show relatively higher connectivity than the lower LAL dominant type. Images are prepared from the data used in Namiki et al. 2014.


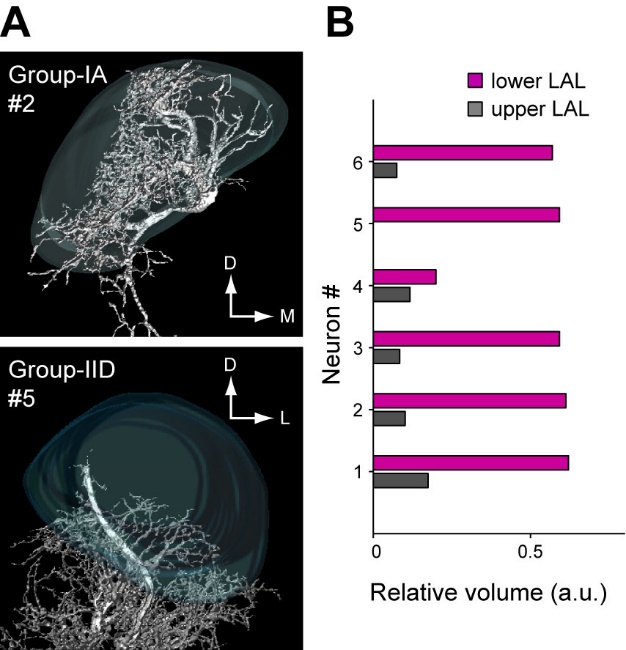


**Supplementary Figure 7| Innervation of the descending neurons in the lateral accessory lobe in the silkmoth.** Relative volumes of the neurite of descending interneurons, which showed the flip-flop response, in the upper (*gray*) and lower divisions of the LAL (*magenta*) (*right*). Two examples of the morphology are shown (*left*). The outer shape of the LAL is shown in color (*blue*). Relative volume was significantly higher for the lower LAL than upper LAL. Images are modified from Namiki et al. 2014.
